# Reduction in N-Acetylglucosaminyltransferase-I Activity Decreases Survivability and Delays Development of Zebrafish

**DOI:** 10.3390/cimb45110575

**Published:** 2023-11-15

**Authors:** M. Kristen Hall, Cody J. Hatchett, Sergei Shalygin, Parastoo Azadi, Ruth A. Schwalbe

**Affiliations:** 1 Department of Biochemistry and Molecular Biology, Brody School of Medicine, East Carolina University Greenville, Greenville, NC 27834, USA; hallma@ecu.edu (M.K.H.); hatchettc14@students.ecu.edu (C.J.H.); 2 Complex Carbohydrate Research Center, University of Georgia, Athens, GA 30602, USA; sergei.shalygin@uga.edu (S.S.); azadi@ccrc.uga.edu (P.A.)

**Keywords:** glycomic profiling, N-glycans, N-acetylglucosaminyltransferase, GnT-I, zebrafish, embryogenesis, brain anlagen, sensory development, motor activity, muscle development

## Abstract

A lack of complex and hybrid types of N-glycans in mice is embryonically lethal due to neural tube maldevelopment. N-acetylglucosaminyltransferase-I (GnT-I; *Mgat1*) catalyzes a required step for converting oligomannose N-glycans into hybrid and complex N-glycans. Unlike mice, zebrafish have two *Mgat1a/b* genes. Herein, CRISPR/Cas9 technology was used to knockdown GnT-Ib activity in zebrafish, referred to as *Mgat1b*^−*/*−^, to examine the impact of a decrease in complex types of N-glycans on survival and development, and sensory and motor functions. Genotyping verified the occurrence of edited *Mgat1b*, and LC-ESI-MS and lectin blotting identified higher levels of oligomannose and lower levels of complex N-glycans in *Mgat1b*^−*/*−^ relative to Wt AB. The microscopic visualization of developmental stages and locomotor studies using an automated tracking unit and manual touch assays revealed reduced survivability, and delayed motor and sensory functions in *Mgat1b*^−*/*−^. Moreover, embryonic staging linked reduced survivability of *Mgat1b*^−*/*−^ to disruption in brain anlagen formation. Birefringence measurements supported delayed skeletal muscle development, which corresponded with motor and sensory function impediments in *Mgat1b*^−*/*−^. Furthermore, GnT-Ib knockdown hindered cardiac activity onset. Collectively, *Mgat1b*^−*/*−^ displayed incomplete penetrance and variable expressivity, such that some died in early embryonic development, while others survived to adulthood, albeit, with developmental delays. Thus, the results reveal that reducing the amount of complex-type N-glycans is unfavorable for zebrafish survival and development. Moreover, our results support a better understanding of human congenital disorders of glycosylation.

## 1. Introduction

Glycosylation, a complex co- and post-translational protein modification, is critical in the development and maintenance of an organism. There are three basic types of N-glycans—oligomannose, hybrid, and complex—each of which share a common pentasaccharide core and are processed sequentially [1]. The various N-glycans result from the addition of different branch points via the action of N-acetylglucosaminyltransferases (GnTs, encoded by *MGAT* genes). The conversion of oligomannose-type *N*-glycans to the hybrid type is initiated via GnT-I (encoded by *MGAT1*), and the hybrid type is further processed to the complex type via GnT-II (encoded by *MGAT2*) [1]. The consequences of aberrant N-glycosylation processing are well noted in the congenital disorders of glycosylation (CDG), an ever-growing disease family that encompasses almost every organ system and often results in neurological complications [2,3].

The magnitude of N-glycan processing in organismal survival and development is well noted in past studies which show that the conditional inactivation of *Mgat1* in neuronal tissue of mice causes severe neurological defects and early post-natal death at about embryonic day E13 [4], while global *Mgat1* knockout mice did not survive beyond E10.5 due to maldevelopment of their neural tubes [5]. Interestingly, although complex N-glycans were observed pre-implantation at around E3.5 in *Mgat1* null embryos, these levels declined by E7.5, thus attributing the earlier levels to maternal derivation and further emphasizing the criticality of complex N-glycans in post-implantation development [6]. Thus, these studies support the importance of complex-type N-glycans in the neurological development of mice. Moreover, expanding the glycan studies to zebrafish is ideal since they have two *Mgat1* genes (*Mgat1a* and *1b*), instead of a single *Mgat1* gene like mice, thus allowing for an examination of how lowered levels of GnT-I activity affect development and survival, minus the lethality associated with mice.

The glycome of zebrafish (*Danio rerio*) is quite diverse, and all of the three N-glycan types are present as early as 6 h postfertilization (hpf), with an abundance of oligomannose-type N-glycans [7]. Although complex-type N-glycans are found in smaller amounts, a sharp increase in levels is noted in the segmentation to the pharyngula period (10–48 hpf) of embryonic development [7]. In adult zebrafish, although the proportion of complex- to oligomannose-type N-glycans varies among specific organs, most of the brain regions revealed a higher population of complex-relative to oligomannose-type N-glycans [8]. Further, complex N-glycans found in the brain of adults were found to have the highest percentage of core fucose and highest overall sialyation pattern relative to other organs [8]. It should also be noted that zebrafish, unlike mice and humans, have two genes (*Mgat1a/b*) that encode GnTIa/b [9]. Thus, the inactivation of one of the *Mgat1* genes will likely result in lowered GnTI activities and thereby decrease the amount of complex-type N-glycans.

Zebrafish is a model organism for vertebrate development due to the rapid progression of developmental stages. Zebrafish development is initiated with a single cell, the zygote period, and progresses to the blastula stage within 3 hpf [10]. It is during the blastula phase when the embryo undergoes several important events, including the start of epiboly, a process of coordinated cell movement critical for body formation [10]. Epiboly continues into gastrulation, during which the yolk is gradually internalized as cells continue to form. This is carried out through the thinning and spreading of the ectodermal layers while the endoderm layers move towards the inside of the embryo [11]. The portion of the yolk cell that is covered by the blastoderm is referred to as percent epiboly, with 75% epiboly occurring at around 8 hpf and 100% epiboly occurring around at 10.5 hpf [10]. During the segmentation phase (10–24 hpf), somites begin to form along with neural keel and neural crest migration. It is during the pharyngula stage (24–48 hpf) when the circulatory system is formed, and a heartbeat is initiated at the onset of this stage [10]. Therefore, by silencing one of the *Mgat1* genes, the development of embryos can be monitored and compared with that in mice with conditional and global *Mgat1* knockouts.

The objective of this study was to create a *Mgat1b* mutant fish strain, *(Mgat1b*^−*/*−^), and to examine the impact of higher levels of oligomannose and less complex N-glycans in the survival and development of *Mgat1b*^−*/*−^ relative to Wt AB zebrafish. Survivability and developmental studies revealed that a reduction in complex-type N-glycans perturbs survivability and development. Embryonic developmental staging implicated disruptions of the brain anlagen, the precursor for brain formation, in the lower survivability of zebrafish with silenced *Mgat1b*. Further, delayed skeletal muscle development coincides with a diminished amount of complex N-glycans, along with a reduction in motor and sensory functions. Likewise, cardiac muscle development is impeded in *Mgat1b*^−*/*−^. Taken together, the results of this study imply that a reduction in the amount of complex-type N-glycans is unfavorable in the survival and development of zebrafish.

## 2. Materials and Methods

### 2.1. Animal Husbandry, Staging, and Larva and Embryo Collections

All Zebrafish procedures were approved by the Institutional Animal Care & Use Committee (IACUC) of East Carolina University. The utilized adult wildtype Pseudoloma-free AB strain (Wt AB) was purchased from Sinnhuber Aquatic Research Laboratory (SARL), propagated at ECU, and utilized for the creation of mutant zebrafish with genetic editing of the *Mgat1b* gene. All zebrafish were maintained at 28 °C on a light/dark cycle of 14 h on and 10 h off in a Pseudoloma-free lab. Embryos collected from natural spawns were transferred to 100 × 15 mm Petri dishes containing egg water (5.03 mM NaCL, 0.17 mM KCl, 0.33 mM CaCl_2_•2H_2_O, 0.33 mM MgSO_4_ •7H_2_O, and 0.05% methylene in 1 L system water) and developed in an incubator set at 28 °C. Embryos were staged based on morphological criteria and time post-fertilization as described by Kimel et al., 1995 [10]. Fish older than 96 h of age were fed a high-protein diet of Gemma Micro pellets (Skretting, Tooele, UT, USA) every morning and afternoon. Larvae (6 dpf and 24 dpf) and adult fish were euthanized using an ice slurry bath for 10 min prior to glycoprotein or RNA analysis. All experiments using live zebrafish were performed at 28 °C. 

The CHOPCHOP program was employed to identify a guide RNA (gRNA) target sequence (ggccggtgaagtgatcagat) in zebrafish *Mgat1b* (accession number: NM_001079971), along with forward (gccatggttcgcaagaaagg) and reverse (ccaattctggtcattgcctg) primers for amplification of the fragment of interest [12,13,14]. The target sequence has a BclI site which is shown in red font. The restriction sequence was selected to identify edited genomic DNA in the target sequence of an individual fish. Oligonucleotides were purchased from Thermofisher, Waltham, MA, USA. The gRNA target sequence also included a T7 promoter sequence added to the 5′ end and a 14 nucleotide overlap sequence added to the 3′ end for use of the EnGen^®^ sgRNA Synthesis Kit, *S. pyogenes* (New England Biolabs, Ipswich, MA, USA). The transcribed nucleotide was purified using Monarch^®^ Kits for RNA Cleanup (New England Biolabs, Ipswich, MA, USA).

### 2.2. Genotyping of Embryos, Larvae, and Adult Fish

After collection of embryos, larvae, or a small portion of the adult fish tail, genomic DNA was extracted by incubating embryos in 50 mM NaOH at 99 °C for 20 min. The NaOH solution containing genomic DNA was used directly for PCR. PCR forward (gccatggttcgcaagaaagg) and reverse (ccaattctggtcattgcctg) primers were designed around the gRNA target and used to amplify the target region. PCR conditions: Initial denaturation for 2 min at 94 °C; 32 cycles of denaturation at 95 °C for 30 s, annealing at 58 °C for 30 s, elongation at 72 °C for 30 s, then a final step at 70 °C for 10 min. Gene editing of the fragment was then identified by restriction enzyme (RE) digest reaction, followed by separation of band(s) on an agarose gel. The presence of an undigested band indicated that the DNA was edited for at least one of the *Mgat1b* alleles.

### 2.3. Generating the Mgat1b^−/−^ Mutant Line

Single-cell embryos were microinjected with an about 500-picoliter mixture solution containing 100 ng/µL of sgRNA and 360 pg/µL of EnGen Spy Cas9 NLS ^®^. Microinjection was driven by compressed N2 gas under the control of a PV820 Pneumatic PicoPump (World Precision Instruments, Sarasota, FL, USA), accomplished using a microcapillary pipette attached to a micromanipulator, under a Nikon microscope (Tokyo, Japan). About thirty microinjected embryos (F0) were collected for estimating gRNA efficiency at 24 h post-fertilization (hpf) via genotyping. If pooled F0 embryos had undigested bands after treatment with the restriction enzyme BclI (Thermofisher, Waltham, MA, USA), then Cas9 successfully cut the targeted region and the cell incorrectly repaired the damage, destroying the BclI site and possibly creating a frameshift mutation. The remaining microinjected F0 embryos were raised to adulthood and then checked for gene editing via fin clipping and restriction enzyme (RE) digest. Adult F0 fish had their DNA sequenced to verify a frameshift, and then, they were outcrossed with Wt AB. F1 embryos were screened for gene editing, and furthermore, mutations were confirmed by DNA sequencing. Notably, the mutation (Δ5) was identical in F0 and F1 adult fish. Fish that were heterozygous for the same mutation were paired for spawning. F2 and embryos from subsequent generations, e.g., F3, were collected and grown into adulthood, when they were screened by RE digestion.

### 2.4. Brain Dissections

Adult zebrafish were euthanized via an ice slurry bath for 10 min following operculum movement cessation in accordance with IACUC protocols. Euthanized fish were placed on a Sylgard (Sigma Aldrich, St. Louis, MO, USA)-coated 100 mm dish. Dissection pins were inserted through the mouth and extended through the ventral side of the fish to affix fish to the dish. Spring scissors were used to cut from the upper lip through the cranium, revealing the brain. Next, two perpendicular incisions were made behind the gills, running dorsal to ventral, starting at the initial cut. The optic nerves and brain stem were severed using spring scissors, and the brain was extracted with forceps. Dissected brains were placed in cryotubes, flash frozen in liquid nitrogen, and stored at −80 °C until ready for use.

### 2.5. Total Brain Membrane Preparations and Larval Homogenate

Total membranes of ten pooled adult zebrafish brains were isolated via ultracentrifugation, as previously described [15]. Total membrane samples for glycan analysis were resuspended in 10 mM Tris, pH 7.4; 250 mM sucrose, 5 mM EDTA; protease inhibitor cocktail set III (Calbiochem, San Diego, CA, USA). All samples were stored at −80 °C until needed. Wt AB and *Mgat1b*^−*/*−^ larval zebrafish were pooled and collected for protein. About 90 larvae (6 dpf and 24 dpf) were resuspended in RIPA buffer (PBS, 1% Triton X-100, 0.5% sodium deoxycholate, and 0.1% SDS) plus protease inhibitor cocktail set III (EMD Biosciences, San Diego, CA, USA), followed by the addition of an SDS-PAGE sample buffer containing 200 mM DTT to reduce and denature the samples for lectin blotting and Coomassie blue staining.

### 2.6. Lectin Blots and Coomassie Blue-Stained Gels

The evaluation of total membrane proteins and larval homogenate was performed via Coomassie staining and lectin blotting. In brief, proteins were allowed to migrate on 10% SDS gels at 20 mA followed by incubation with Coomassie^®^ Brilliant Blue (MP Biomedical, Solon, OH), or transferred to nitrocellulose membranes (Whatman, Dassel, Germany) at 250 mA for lectin blotting, as previously described [15]. Transferred proteins were probed with biotin-conjugated E-PHA or GNL lectins (10 µg/mL; Vector Laboratories, Burlingame, CA, USA).

### 2.7. RNA Extraction

Approximately 95 larval zebrafish were collected in a microcentrifuge tube placed in an ice slurry and resuspended in TRIzol ^®^ (Invitrogen, Carlsbad, CA, USA) and homogenized until sufficiently disrupted. Homogenized samples were allowed to sit at room temperature for five minutes to complete dissociation of samples, followed by the addition of chloroform. Samples were centrifuged at 12,000× *g* for 5 min at 4 °C. The upper aqueous phase was collected, RNA was precipitated via the addition of isopropanol, and the RNA pellet was washed with ethanol. Total RNA was resuspended in RNAse-free water. Total RNA cleanup was performed using the RNeasy mini kit (Qiagen, Hilden, Germany) per the manufacturer’s protocol. The RNA quality and quantity was assayed using a NanoDrop 1000 (ThermoFisher Scientific, Waltham, MA, USA).

### 2.8. RT-PCR

A total of 1 µg of RNA in a 20 µL reaction was reverse-transcribed utilizing the iScript™ cDNA Synthesis Kit (Bio-Rad Laboratories, Hercules, CA, USA) according to manufacturer’s instructions. Quantitative real-time PCR was performed with a CFX96 Touch Real-Time PCR Detection System using IQ™SYBR Green SuperMix (Bio-Rad Laboratories, Hercules, CA, USA) and primers from ThermoFisher Scientific, Waltham, MA, USA. Primers for amplification were designed so the products would span at least one intron using Primer-Blast [16], except those for elfα [17]; see Table S1 for sequences. The amplification of a single product was confirmed via agarose gel visualization and/or melting curve analysis for all primer sets; see Figure S3. mRNA was quantified via ΔΔCT analysis using housekeeping genes β-actin and ELFα for normalization.

### 2.9. N-Glycan Analysis

Total membrane protein fractions from the brain of Wt AB and *Mgat1b*^−*/*−^ adult zebrafish were diluted with 100 µL of 50 mM ammonium bicarbonate buffer, followed by the addition of 25 µL of 25 mM Dithiothreitol (DTT) and incubated at 50 °C for 30 min [18]. The samples were subsequently desalted by centrifugal filtration using 10 k Amicon centrifuge filters (MilliporeSigma, Burlington, MA, USA, Cat. No. UFC501096) and then ultrasonicated to dissolve the proteins. The N-glycans were released from the samples by adding 5 µL of PNGaseF (New England Biolabs, Ipswich, MA, USA, Cat. No. P0709L) and incubating at 37 °C for 48 h. The released N-glycans were permethylated by using methyl iodide in the presence of NaOH-DMSO base. The permethylation reaction was quenched by the addition of water, and the permethylated N-glycans were extracted with dichloromethane. The dichloromethane layer was washed with water four times and evaporated to dryness by nitrogen gas, and the permethylated N-glycans were mixed in a 1:1 methanol–water (plus 1 mM NaOH) mixture for the LC-ESI-MS/MS analysis. Samples were analyzed on an Orbitrap Fusion Tribrid mass spectrometer equipped with a nanospray ion source and connected to a nanoLC system (Thermo Ultimate 3000). A nano-LC column 15 cm in length with 75 µm internal diameter filled with 3 µm C18 material (reverse phase) was used for chromatographic separation of the glycans. LC-ESI-MS/MS runs were performed for 72 min gradients using a sodiated buffer system (Buffer A: 1 mM NaOAc in H_2_O; Buffer B: 80% ACN, 0.1% formic acid, and 1 mM NaOAc). A precursor ion scan was acquired at 120,000 resolution in the Orbitrap analyzer, and precursors at a time frame of 3 s were selected for subsequent MS/MS fragmentation in the Orbitrap analyzer at 15,000 resolution. MS/MS fragmentation was conducted with fixed CID (collision energy 40%) using a data-dependent scan (DDS) program, which performs an MS/MS acquisition for the most abundant ions in the MS spectrum. Precursors with an unknown charge state or a charge state of +1 were excluded, and dynamic exclusion was enabled (30 s duration). LC-MS/MS data were analyzed using Thermo Xcalibur 4.2, Thermo FreeStyle 1.8 SP2, and GlycoWorkBench 2.1 software. The area under the curve of different charge states of each N-glycan peak were extracted from the LC-MS chromatogram and added, and the relative percentages of individual N-glycans were calculated.

### 2.10. Tactile-Evoked Escape Response

A touch-evoked escape response assay was performed with embryos and larvae at 2 and 3 dpf, respectively, (fish that appeared maldeveloped were not used) from male and female crossed using Wt AB or *Mgat1b*^−*/*−^. Two dpf fish were manually dechorionated using needles, as needed. After at least 1 h, 15 larvae were put in a 100 mm dish with egg water and allowed to acclimate on a stereoscope for 10 min. A tactile stimulus was administered by a gentle tap with a P10 micropipette tip to the tail of the larvae. The escape behavior was scored according to the number of taps it took to get the larvae to swim away (response). All-points histograms were used to show the total number of larvae that responded to 1-9 taps and ≥10 taps. The mean ± S.D. was also determined from the total number of larvae tested for both groups and 2 and 3 dpf. 

### 2.11. Vibrational Startle Response

Wt AB and *Mgat1b*^−*/*−^ zebrafish larvae (10 dpf) were placed individually into a well of a 6-well plate containing roughly 4 mL of system water and then placed in the chamber of the Zantiks MWP unit. An acclimation period of 5 min was followed by a 20 s tracking period to locate the fish. Pre-startle activity was then logged for 10 s, followed by a 1 s vibration. The machine then tracked post-startle for 20 more seconds. The vibration and post-startle were repeated 2 more times, with the combined startle activity averaged. 

### 2.12. Swimming Locomotor Activity

Wt AB and the *Mgat1b* homozygous mutant were maintained in 100 mm dishes containing egg water at 28 °C until 5 dpf, at which point the larvae (≥12) were housed in a 250 mL beaker containing a mixture of egg water and system water. The ratio of egg water to system water was adjusted incrementally as follows: 5 dpf (75:25), 6 dpf (50:50), 7 dpf (25:75), and ≥8 dpf (0:100). At 14 dpf, the larvae were transferred to a 1.8 L tank containing system water. For motor activity recordings, a single fish was placed in one well of a six-well plate containing 11 mL of egg water. The plate was transferred to the MWP unit (Zantiks, Cambridge, UK), and the fish was allowed to acclimate for 5 min. The motor activity of the larvae was tracked for five minutes (one minute intervals), with each one-minute interval followed by a 60 s acclimation period and a 10 s target acquisition period. The protocol was run in triplicate for each plate. After the procedure, the fish were removed from the plate and transferred to a clean beaker or tank containing a clean larvae medium and returned to the incubator. This process was repeated on the following days post-fertilization (dpf): 5, 7, 9, 14, 24, and 29.

### 2.13. Skeletal and Cardiac Muscle Developmental Assays

The skeletal muscle integrity of Wt AB and *Mgat1b*^−*/*−^ fish strains was monitored in embryos (58 hpf) and larvae (3 dpf and 4 dpf) via the employment of a birefringence assay [19]. The 58 hpf embryos were dechorionated with needles, if needed. Images were collected using a Leica M80 stereoscope and an attached Leica IC90 E camera at 2× magnification with two polarized lenses. One lens was stationary, and the other was rotated to adjust the polarizing light for muscle fiber illumination. Using image J, mean intensity measurements were taken from somites 12 through 20. The birefringence was normalized to Wt AB for each timepoint. Heart development was assayed by the direct visual observation of a heartbeat in zebrafish embryos at 25 hpf and 28 hpf using a Zeiss 47 50 52- 9901 stereoscope at 5× magnification. The number of embryos with a visually detectable heartbeat was recorded as the percent per plate. Experiments were set up on 2 occasions. Five and seven clutches were collected for Wt AB and *Mgat1b*^−*/*−^. Twenty embryos were added to each plate for Wt AB, and eight, sixteen, fifteen, ten, twenty-two, twenty-five, and thirty-two embryos were added per plate for Mgat1b^−/−^. The percentage of embryos with a heartbeat was determined per plate. 

### 2.14. Statistical Analysis

The N-glycan structures were assigned with the aid of GlycoworkBench 2.1 software based on precursor masses (sodium adduct) obtained by ESI-MS/MS and the common mammalian biosynthetic pathway [18]. Image J 1.54d software was used for somite mean intensity measurements. Adobe Photoshop was employed to obtain agarose gel and lectin blot pictures. Origin 9.55 was used for graphics and statistics. Data are presented as the mean ± S.E., where n denotes the number of observations, as indicated. A statistical comparison of two groups was accomplished using unpaired Student’s *t*-test.

## 3. Results

### 3.1. Generating the Mgat1b^−/−^ Mutant Line

**Figure 1:**
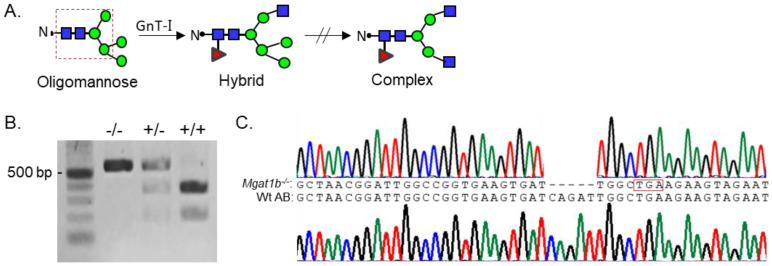
Generation and classification of the *Mgat1b*^−*/*−^ mutant line. Depiction of N-glycan types and the sequential processing initiated via GnT-I (*Mgat1*) from the simplest N-glycan type (oligomannose) to more processed hybrid and complex types. The area encased within the dashed red box indicates the common pentasaccharide core (**A**). Products of BclI digest, which yield either homozygous *Mgat1b*^−*/*−^, heterozygous *Mgat1b^+^*^/−^, or Wt AB (*Mgat1b^+/+^*). Number adjacent to Kb ladder (500 bp) indicates band size (**B**). Chromatogram with coding sequence of a fragment of Mgat1b from a Wt AB fish compared to that of *Mgat1b*^−*/*−^, revealing five nucleotide deletions (Δ5), which generated a premature stop codon (encased in red box) (**C**).

Since *Mgat1* (codes GnT-I) inactivation in mice was embryonically lethal due to defects in neural tube formation, we employed zebrafish as a model to examine whether reduced levels of complex N-glycans would disrupt development. Although zebrafish synthesize two N-acetylglucosaminyltrases (GnT-Ia and Ib) while mice and humans have a single GnT-I [1], zebrafish provides an excellent and useful model since we could introduce a premature termination codon in one *Mgat1* gene (GnT-Ib), which enabled us to assess the impact of reduced GnT-I activity in both embryogenesis and development to adulthood, minus the lethality that is observed early in mice. These enzymes catalyze the conversion of oligomannose-type N-glycans to hybrid type N-glycans which in turn generates complex-type N-glycans (Figure 1A) [1]. GnT-Ib is a type II single-pass transmembrane protein, composed of 524 amino acid residues, and resides in the Golgi apparatus [20,21]. Further, the catalytic domain is found in the C-terminal region of the protein [20]. Adult fish with an indel in the *Mgat1b* were identified by restriction enzyme digestion of an amplified fragment of the coding sequence (CDS) of *Mgat1b* (Figure 1B). The fragment in Wt AB contained a BclI site, as the digested amplified band produced two fragments, while the mutant fish (*Mgat1b*^−*/*−^) generated a single larger band. Fish that were heterozygous for the mutation produced three bands, representing Wt and mutant *Mgat1b* alleles. DNA sequencing of the mutant zebrafish line revealed five deleted nucleotides (Δ5) which generated a premature stop codon (Figure 1C). This deletion would result in a C-truncated protein of 70 amino acid residues lacking catalytic activity [20]. Two N-truncated proteins of 381 and 377 residues in length could potentially be generated via two Met residues at positions 144 and 148. However, these N-truncated proteins would not be localized to the Golgi apparatus, since GnT-I is localized via its transmembrane segment [21].

### 3.2. Glycomic Profiling of Wt AB and Mgat1b^−/−^ Fish Lines Reveals Different Levels of Complex, Hybrid, and Oligomannose Types of N-Glycans

**Figure 2:**
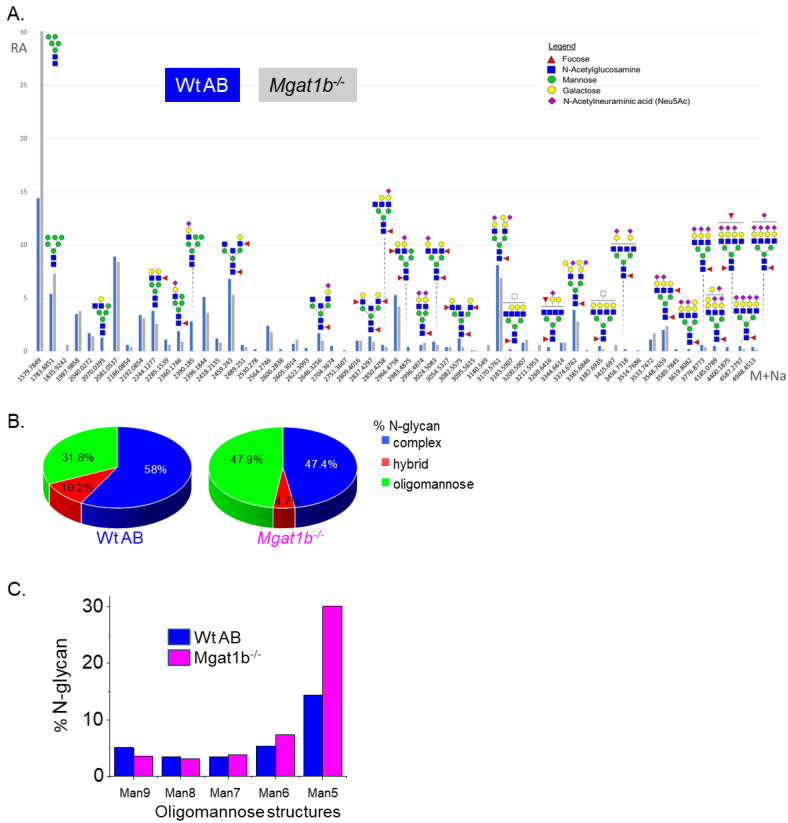
Comparison of LC-ESI-MS relative intensities of the permethylated N-glycans derived from Wt AB and the *Mgat1b*^−*/*−^ mutant line. LC-ESI-MS intensities of released N-glycans from Wt AB (blue bars) and *Mgat1b*^−*/*−^ (grey bars) (**A**). All molecular ions are present in sodiated form ([M + Na]+). N-glycan numbers correspond to Figures S1 and S2, and Tables S2 and S3. Breakdown of the percent of various detected N-glycan types (**B**) and types of oligomannose structures (**C**) identified by the ESI-MS spectra.

To identify specific glycan structures, isolated permethylated N-glycans from the adult brains of Wt AB and *Mgat1b*^−*/*−^ were analyzed by LC-ESI-MS. In both cases, detectable levels of the oligomannose, hybrid, and complex types of N-glycans were observed (Figure 2A). However, higher levels of Man5 and Man6 oligomannose N-glycans and lower levels of hybrid and complex types of N-glycans were in *Mgat1b*^−*/*−^ relative to Wt AB. In both fish strains, the levels of oligomannose and complex types of N-glycans were greater than 31%, while that of the hybrid type were much lower, ranging from 5 to 10% (Figure 2B). The rise in the oligomannose type corresponded to decreases in both complex and hybrid types. When comparing the levels of Man9 to Man5 oligomannose structures between *Mgat1b*^−*/*−^ and Wt AB, there was about two-fold greater level of Man5 in the mutant line (Figure 2C). The accumulation of Man5 strongly supports that *Mgat1b* is at least partially inactive as this is the substrate utilized by GnT-Ib. Thus, the glycan population of adult brain from *Mgat1b*^−*/*−^ fish has less complex-type N-glycans than Wt AB fish.

### 3.3. Increased Levels of Oligomannose N-Glycans and Decreased Mgat1b Expression in Mutant Strain of Adult and Larvae Fish

**Figure 3:**
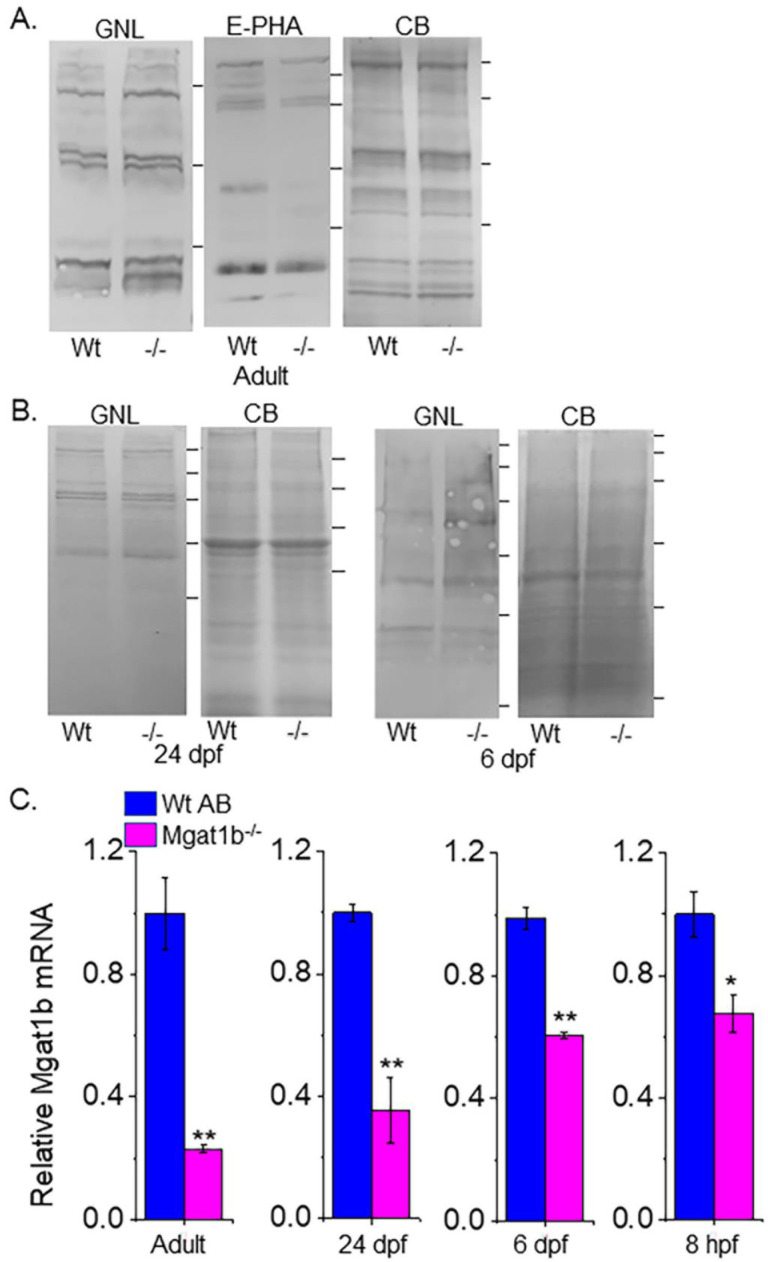
Confirmation of decreased Mgat1b expression, and an increase in oligomannose N-glycans in mutant strain of adult and larvae fish. Lectin blots of whole cell lysates of adult brain (**A**), and larvae at 24 dpf and 6 dpf from Wt AB and Mgat1b^−/−^ mutant zebrafish (**B**). Separated proteins were probed with Phaseolus vulgaris Erthroagglutinin (E-PHA) or Galanthus nivalis lectin (GNL). Coomassie blue (CB)-stained gel demonstrating equal protein loads among the samples. Lines adjacent to the blots denote protein markers (in KDa) 250, 150, 100, 75, 50, 37. Bar graphs represent Mgat1b mRNA levels from Wt AB and Mgat1b^−/−^ mutant zebrafish relative to housekeeping genes β-actin (**C**) and ELFα (Figure S1). Data are presented as mean ± SEM, *n* ≥ 3 and were all compared using Student’s *t*-test (** *p* < 0.005, * *p* < 0.02).

To verify that GnT-Ib activity was minimal in the adult brains (Figure 3A), as well as 24 dpf and 6 dpf larvae (Figure 3B) of *Mgat1b*^−*/*−^ relative to Wt AB, glycosylated proteins from whole cell lysates of each strain were separated on a reducing SDS gel and probed with *Galanthus nivalis* lectin (GNL). This lectin has higher affinity for the oligomannose type than the hybrid or complex types of N-glycans [22]. The overall band intensities of glycosylated proteins from the adult brains of *Mgat1b*^−*/*−^ were higher than those from Wt AB. Moreover, less *Phaseolus vulgaris* Erythoagglutinin (E-PHA), a lectin with very high affinity for complex-type N-glycans with bisecting N-acetylglucosamine (GlcNAc) relative to other N-glycan structures [22], bound to glycosylated proteins from the adult brain of *Mgat1b*^−*/*−^ more often than that of Wt AB. GNL also had a preference for binding to glycosylated protein from whole larvae (24 dpf and 6dpf) of *Mgat1b*^−*/*−^ over those from Wt AB. In all cases, the amount of protein loaded for each sample was detected with Coomassie blue-stained SDS gels, indicating comparable levels of protein. 

Since the *Mgat1b* gene had an indel in the CDS and oligomannose N-glycans were reduced, it is likely that the abundance of the *Mgat1b* transcript is lowered in fish from *Mgat1b*^−*/*−^ relative to Wt AB. The qRT-PCR assay showed significantly lower levels of *Mgat1b* mRNA in adult brains from *Mgat1b*^−*/*−^ using β-actin (Figure 3C) or ELFα (Figure S1) as housekeeping genes. The levels of *Mgat1b* mRNA were also reduced in whole larvae zebrafish from 24 dpf and 6 dpf, as well as embryos at 8 hpf of the mutant strain. The reduction in *Mgat1b* transcripts was less in the entire organism than in the brain and, furthermore, was less reduced at the earlier post-fertilization timepoints. This decay in *Mgat1b* mRNA throughout zebrafish development may be due to the non-sense mediated decay mechanism [23], as Mgat1b gene editing resulted in a premature termination codon. Taken together, the results indicate that *Mgat1b*^−*/*−^ fish have reduced GnT-I activity, and therefore, zebrafish from the *Mgat1b*^−*/*−^ strain have higher amounts of oligomannose N-glycans and lower levels of complex N-glycans. 

### 3.4. Low Survival of Embryonic Mgat1b^−/−^ Mutants after Reaching 75% Epiboly

**Figure 4:**
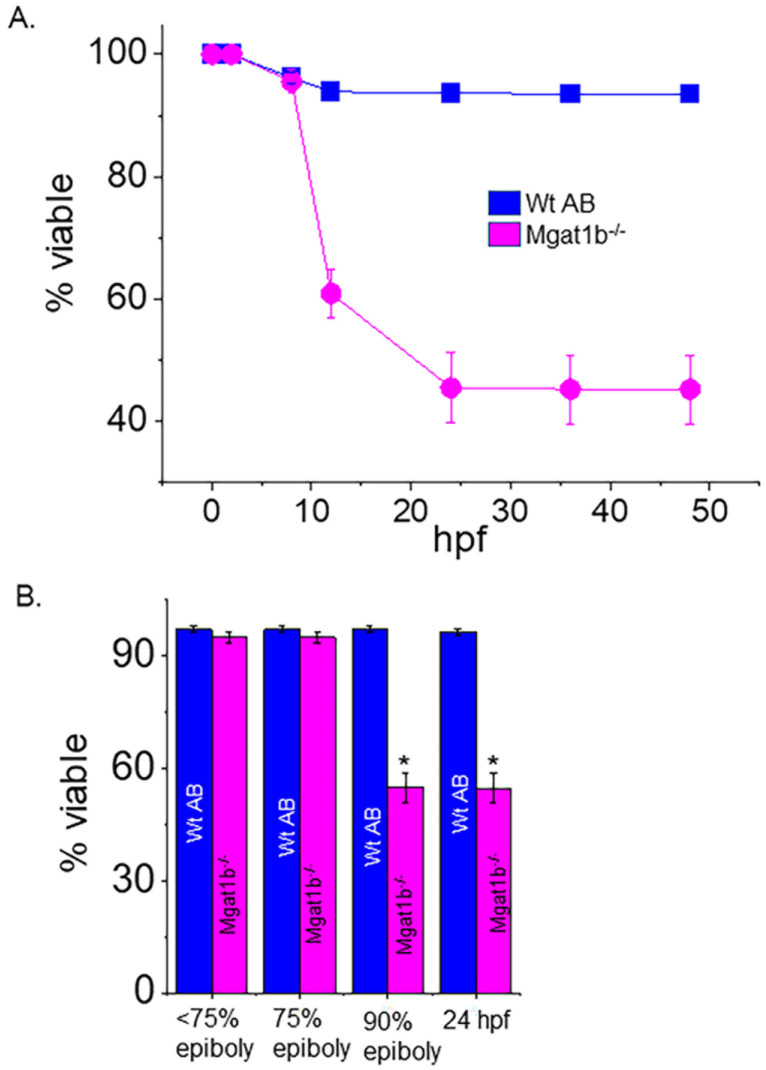
Decline in survivability of Mgat1b^−/−^ mutants post 75% epiboly relative to Wt AB. Survivability of Wt AB and *Mgat1b*^−*/*−^ zebrafish tracked up to 48 hpf (**A**). Percent of viable embryos at the various stages of embryogenesis (**B**). Data are presented as mean ± SEM, *n* ≥ 8 clutches, and were all compared using Student’s *t*-test (* *p* < 0.0001).

Survival of the *Mgat1b*^−*/*−^ and the Wt AB fish was tracked up to 72 hpf. *Mgat1b*^−*/*−^ survival was largely lowered at 12 hpf, and a smaller decrease was detected at 24 hpf while only minimal decreases in survival were observed after this time (Figure 4A). Next, we monitored the various stages of embryogenesis for *Mgat1b*^−*/*−^ and Wt AB (Figure 4B). By far, the majority of the Wt AB offspring survived while only about half of the *Mgat1b*^−*/*−^ embryos survived prior to reaching 90% epiboly. As such, most death from *Mgat1b*^−*/*−^ embryos occurred during the transition from 75% epiboly to 90% epiboly, a period heavy in coordinated cell migration, supporting that increased oligomannose-type N-glycans may hamper cell migration, thus proving to be detrimental in the progression of the later stages of epiboly.

### 3.5. Motor and Sensory Functions Were Hampered in Mgat1b^−/−^ Mutants

**Figure 5:**
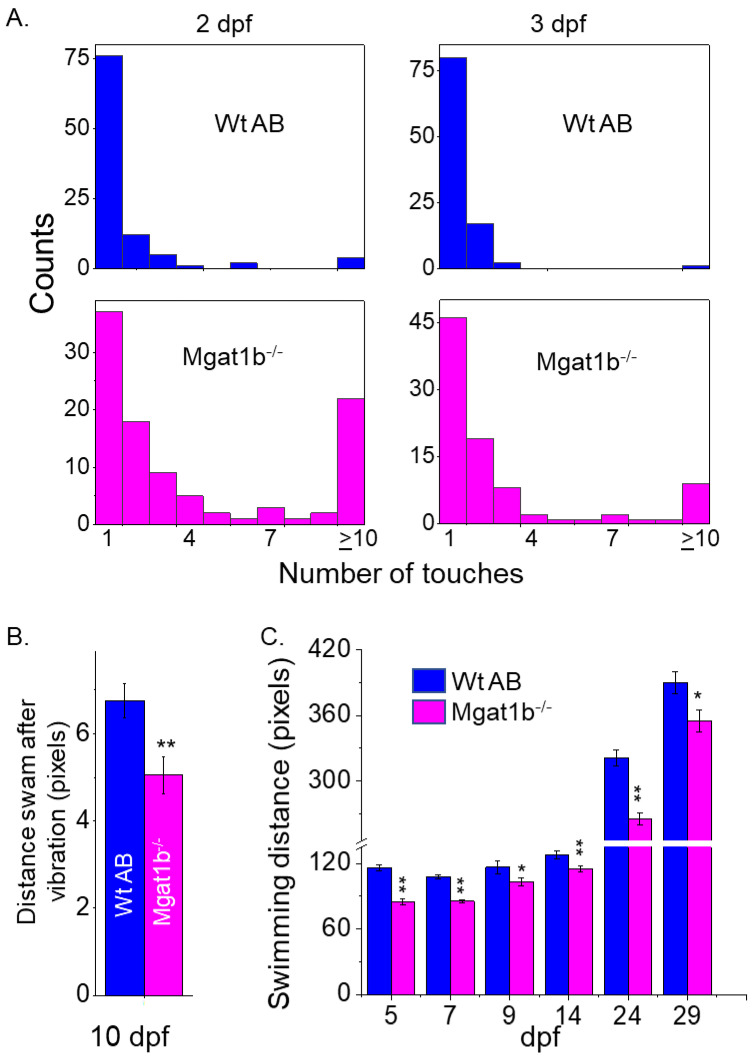
Inhibition of motor and sensory functions in *Mgat1b*^−*/*−^ mutants. Touch-evoked response of Wt AB (upper panels) and *Mgat1b*^−*/*−^ (lower panels) at 2 and 3 dpf, as indicated (**A**). Data are presented as mean ± SEM, *n* ≥ 90 larvae. Distance swam in response to vibrational stimuli exhibited in 10 dpf larvae, *n* ≥ 45 larvae (**B**). Spontaneous locomotor swimming distance recorded in 5–29 dpf larvae, *n* ≥ 6 fish (**C**). Data are presented as mean ± SEM and were compared using Student’s *t*-test (** *p* < 0.005, * *p* < 0.05).

Since the inactivation of *Mgat1* in mice had dramatic effects on neural structure [4], the motor–sensory function of *Mgat1b*^−*/*−^ embryos and larvae zebrafish was compared to those of Wt AB. The touch-evoked escape response assay was conducted to simultaneously determine the ability of Wt (2 dpf, 1.71 ± 1.92, *n* = 100; 3 dpf, 1.3 ± 0.99, *n* = 100) and mutant (2 dpf, 4.03 ± 3.63, *n* = 100; 3 dpf, 2.76 ± 2.90, *n* = 90) larval zebrafish to sense tactile stimuli on their tail region and to respond via locomotor activity (Figure 5A). Over 75% of Wt AB swam away immediately (1 touch) at 2 and 3 dpf. Less than 40% responded immediately for *Mgat1b*^−*/*−^ at 2 dpf but the response increased to about 50% at 3 dpf. *Mgat1b*^−*/*−^ larval zebrafish of 10 dpf also showed a decrease in the distance swam in response to vibration stimuli compared to those of Wt AB (Figure 5B). When observing spontaneous locomotor swimming from 5 dpf to 29 dpf, the *Mgat1b*^−*/*−^ larval zebrafish swam shorter distances than the Wt AB (Figure 5C). Notably, larvae from the *Mgat1b^+/^*^−^ mutant line had similar swimming locomotor activities to the *Mgat1b^+/+^* and Wt AB lines, while that of the *Mgat1b*^−*/*−^ line was different, indicating that off-target effects were negligible; see Figure S4. Taken together, these results showed that the mutant embryos and larvae had impaired motor and sensory functions, demonstrating that diminished complex-type N-glycans causes sensory and movement inhibition and thus are critical components in zebrafish survival.

### 3.6. Delayed Development of Skeletal and Cardiac Muscle in Mgat1b^−/−^ Mutants

**Figure 6:**
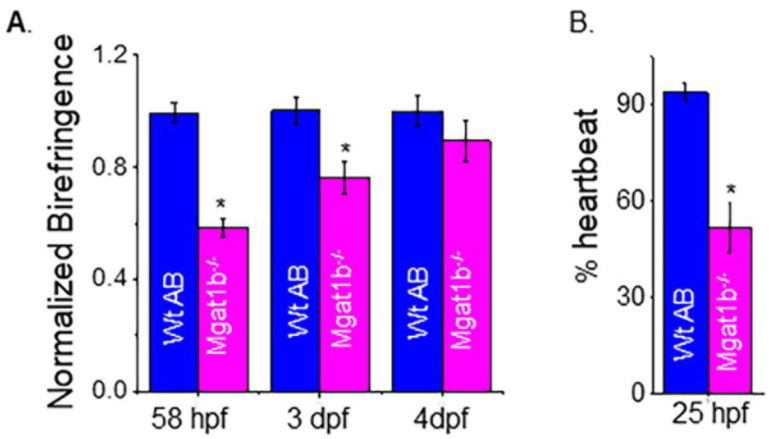
Maldevelopment of skeletal and cardiac muscle in *Mgat1b*^−*/*−^ mutants. Normalized birefringence of Wt AB (*n* = 26, 31, 22 as 58 hpf, 3 dpf, and 4 dpf) and *Mgat1b*^−*/*−^ (*n* = 30, 25, and 22, at 58 hpf, 3 dpf, and 4 dpf); *n* denotes number of examined embryos or larvae (**A**). Percent of Wt AB and *Mgat1b*^−*/*−^ zebrafish with detected heartbeat at 25 hpf, *n* = 5 and *n* = 7 for Wt AB and *Mgat1b*^−*/*−^, respectively (**B**). Data are presented as mean ± SEM and were compared using Student’s *t*-test (* *p* < 0.003).

To examine muscle development, we evaluated skeletal muscle sarcomere organization and when heartbeat could be detected. Light intensity was lower in mutant embryo zebrafish at 58 hpf and larval zebrafish at 3 dpf and 4 dpf than in Wt AB, as indicated when birefringence was normalized to Wt AB (Figure 6A). The inhibition of light emitted is indicative of disorganized skeletal muscle; more organized tissue allows for more light emission. The birefringence for Wt AB was similar over the covered period while mutant fish had an increase and approached that of the Wt AB larval fish over the time course. The occurrence of a heartbeat in Wt AB embryos was greater at 25 hpf (90%), while it was about 50% in *Mgat1b*^−*/*−^ embryos (Figure 6B). In both cases, all embryos had heartbeats at 28 hpf. Thus, these experiments support that skeletal and cardiac muscle development is hampered in the *Mgat1b*^−*/*−^ zebrafish, signifying the role of complex-type N-glycans in muscle formation. 

## 4. Discussion

Previously, our lab utilized in vitro cell models to demonstrate that disruption in the N-glycosylation pathway alters aberrant cellular properties in neuronal-derived cells [15,24] and that the ecotopic expression of incomplete N-glycosylation of a voltage-gated K^+^ channel (Kv3.1b) in primary motor neurons (e.g., caudal primary, CaP) of zebrafish results in maldeveloped CaP neurons [25]. Here, we extended those studies to reveal the deleterious consequences of a global knockdown of one of two GnT-I enzymes (GnT-Ib) in zebrafish. Genotyping confirmed an introduced frameshift in the *Mgat1b*^−/−^ strain. Additionally, swimming locomotor activity in the *Mgat1b*^−/−^ larvae was slower than that of Wt AB, as well as *Mgat1b*^+/+^ and *Mgat1b*^+/−^, minimizing the likelihood of off-target effects. The glycomic analysis supported an increase in the oligomannose type, along with decreases in the hybrid and complex types of N-glycans in the adult brains of the *Mgat1b*^−/−^ zebrafish strain compared to the Wt AB strain. Lectin blotting and qRT-PCR supported the knockdown in GnT-I activity in the adult brain, as well as the whole organism from 8 hpf to 24 dpf. Although prior glycomic studies have reported on the overall glycan populations of embryos and various zebrafish organs [7,8], our study investigated how reduced GnT-I activities contribute to the N-glycan population by altering the various types of N-glycans, such as lowering the levels of complex-type N-glycans. Moreover, our study investigated the negative consequences in the survival and development of zebrafish from this change in the N-glycan population.

N-glycomics is an important analytical technique that has been used in numerous different aspects of research to correlate glycan structure with cellular function [15,26,27]. Assaying *Mgat1b*^−/−^ in conjunction with Wt AB revealed that reduced complex-type N-glycans decreased the survivability of the mutant fish and, furthermore, delayed embryonic development. Embryonic survivability was about 50% for the *Mgat1b*^−*/*−^ mutant strain. To elaborate, there was a drastic decline in embryonic survivability from 8 to 12 hpf and less decline after 12 hpf for the *Mgat1b*^−*/*−^ mutant strain. Further, various clutches had a similar survivability as the median value of percent viable embryos was like the mean value. Since embryonic development relies on maternal gene products [28], it is of interest to evaluate the contribution of maternal mRNA. Our observations of developmental staging revealed that the *Mgat1b*^−*/*−^ mutant fish were lagging in development as they reached 75% epiboly at about 9 hpf and those that survived reached 90% epiboly at about 10–11 hpf, while the Wt AB embryos followed the developmental staging outlined by Kimmel et al., 1995 [10]. As such, the majority of *Mgat1b*^−*/*−^ embryos died during the transition from 75% to 90% epiboly, a stage of prominent cell movement, which transpires around 8–9 hpf in Wt AB zebrafish [10]. Given that at 90% epiboly, brain anlagen formation occurs [29], it is plausible to associate the inability of the *Mgat1b*^−*/*−^ to transition from 75% to 90% epiboly with inefficient cell migration to the lessened complex-type N-glycans. Interestingly, the embryonic development of zebrafish at 12–15 hpf is equivalent to 9.5 to 10.5 embryonic days in mice [30], at which point GnT-I knockout is lethal due to maldevelopment of the neural tube [5], indicating that complex-type N-glycans are crucial even earlier in zebrafish development relative to mice since we observed a drastic decline in survival prior to 12 hpf. Taken together, our current study implicates GnT-Ib knockdown as an impediment in zebrafish embryonic survivability and, specifically, supports that the developmental progression is halted due to possible inhibition of brain anlagen formation.

Complex-type N-glycans are present before brain anlagen development and during neuron development. Reports have shown that all three glycan types form as early as 6 hpf [7]. Moreover, glycomic studies indicated variable expression levels of complex-type N-glycans and a significant increase in the complex type at 12–15 hpf, [30], which is during the segmentation period when somites are formed and during the initiation of primary organs [10]. Our past studies supported the involvement of complex N-glycans in cell migration [15,24,25] and, further, revealed the impact of partially N-glycosylated Kv3 channels, containing Kv3.1b protein, on primary motor neuron development and swimming locomotor activity in zebrafish [25]. Together, the necessity of complex-type N-glycans is evident in embryonic development and neuronal function of zebrafish.

For the surviving *Mgat1b*^−*/*−^ zebrafish, the influence of GnT-I knockdown in skeletal muscle organization and function was visible in the locomotion assays. Muscle performance during burst movements upon sensing external stimuli [19,31,32] was impaired at 2, 3, and 10 dpf in mutant fish relative to Wt AB. Further, spontaneous swimming distance, which is indicative of sustained muscle activity [32], was quite diminished in the *Mgat1b*^−*/*−^ mutant compared to Wt AB from 5 to 29 dpf. Organization of the muscle sarcomeres increased during skeletal muscle development [19,31]. Here, we show that the organization of the sarcomeres was reduced in the embryos and larvae with GnT-I activity knocked down. Thus, the delay in skeletal muscle development coincides with a reduction in motor and sensory functions. Likewise, past studies using the conditional inactivation of *Mgat1* in mice corroborates the relevance of complex-type N-glycans in locomotor activity [4]. As such, our study implicates that inadequate levels of complex-type N-glycans can impair skeletal muscle development, thus coinciding with less efficient locomotor activity in zebrafish.

A range of diverse phenotypes from no marked clinical phenotype to severe disease is found in individuals with similar genetic variants [33]. Here, the indel introduced in both alleles of *Mgat1b* showed incomplete penetrance and variable expressivity. As noted, embryonic survivability and developmental delays, including cardiac and skeletal muscle development, were observed in *Mgat1b*^−*/*−^ fish. Moreover, sensory and motor developmental delays were observed at 48 hpf in *Mgat1b*^−*/*−^ fish as less than 40% of embryos showed burst movements in response to one touch, while 65% of Wt AB had a response. Developmental delays in sensory and motor functions of the *Mgat1b*^−*/*−^ larvae were also noted at 3 dpf, which was supported by the delay in skeletal muscle development. Continued measurements of muscle performance via burst movements upon sensing external stimuli [19,32] and sustained muscle activity [32] revealed delays in motor function and sensory physiology throughout larval development. Thus, fish homozygous for the *Mgat1b* mutation showed different degrees of disease, which support individuals suffering from congenital disorders of glycosylation, including disorders of N-glycosylation processing [2].

## 5. Conclusions

Defective N-glycan processing is a known deterrent in organismal survival and development, as noted in congenital orders of glycosylation [2]. The impact of this rapidly growing disease family is devastating as it spans most organs and systems, yet minimal treatment options are available. Our study utilizing zebrafish reveals that increased oligomannose-type N-glycans concomitantly with reduced complex-type N-glycans diminishes overall survival, while those surviving exhibit significant developmental delays. It is of interest to note that the developmental delays traverse the nervous, cardiovascular, and skeletal systems. Moreover, developmental staging herein affords the opportunity to perhaps pinpoint specific periods of development in which certain N-glycan populations are crucial, particularly in terms of organ development. As such, these results suggest glycan populations as potential diagnostic targets/therapeutic options for a variety of human diseases, including congenital defects to adult onset. Our future direction is to engineer additional *Mgat1* glycosylation mutant zebrafish strains, to perform similar developmental and behavioral studies, and to extend more diverse behavioral studies to juveniles and adults. Moreover, we will expand studies to include spinal cord morphology and microscopic evaluations of the neuronal structure, with the intent to corroborate the influence of complex-type N-glycans in organismal survival, development, and behavior. Although glycan studies that relate to survival and development in zebrafish are relatively minimal and we acknowledge our study could be enhanced by the creation of additional N-glycan knockout strains, as well as by performing a glycomic analysis of other tissues/organs, we believe that our study contributes to a better understanding of specific N-glycan populations in organismal development and function.

## Data Availability

The data are available upon request.

## Supplementary Materials

The following supporting information can be downloaded at https://www.mdpi.com/article/10.3390/cimb45110575/s1, Table S1. Zebrafish primers used for real-time PCR; Table S2. Relative abundancies of each of the N-glycans from the Wt AB strain using ESI-MS; Table S3. Relative abundancies of each of the N-glycans from the Mgat1b−/− strain; Figure S1. LC-ESI-MS intensities of the permethylated N-glycans derived from the Wt AB zebrafish strain; Figure S2. LC-ESI-MS intensities of the permethylated N-glycans derived from the Mgat1b−/− zebrafish strain; Figure S3. Comparing relative levels of Mgat1b transcripts from adult Wt AB and Mgat1b−/− zebrafish strains. Transcript levels were normalized to elfa (A). RT-PCR products run on agarose gel (B). The sizes of the fragments were as follows: Mgat1b, 129 bp; β-actin, 95 bp; elfa, 358 bp; Figure S4. Swimming locomotor activity of Wt AB and the various Mgat1b zebrafish strains. Data are presented as mean ± SEM, n ≥ 21 and were compared by one-way ANOVA with Holmes–Bonferroni adjustments, * *p* < 0.05).
