# Supplementary material for: Reduction in N-Acetylglucosaminyltransferase-I Activity Decreases Survivability and Delays Development of Zebrafish
Source: Curr Issues Mol Biol. 2023 Nov 15;45(11):9165–80. doi: 10.3390/cimb45110575 (PMC10669939; doi:10.3390/cimb45110575)
Supplement: Supplementary file 1 [file cimb-45-00575-s001.zip › cimb-2701579-supplementary.pdf]

Table S1. Zebrafish primers used for real time PCR.

Gene	Forward	Reverse
Mgat1a	GGTTGTTGTTGTGGAGGACGA	TTGTCATTCCAGGCAGACACG
Mgat1b	ACAAGATCGCCAGGCACTAC	ACAGCGCACGGAAATACTCA
β-actin	AGTTGTCTAACAGGGGAGAGC	TTGTGAGGAGGGCAAAGTGG
elfα	CTTCTCAGGCTGACTGTGC	CCGCTAGCATTACCCTCC

Table S2. Relative abundancies of each of the N-glycans from the Wt AB strain using ESI-MS.

Observed m/z (Na^+)	Composition	Relative %
1579.7849	$(Hex)_2 + (Man)_3(GlcNAc)_2$	14.4
1783.8851	$(Hex)_3 + (Man)_3(GlcNAc)_2$	5.4
1987.9858	$(Hex)_4 + (Man)_3(GlcNAc)_2$	3.5
2040.0272	$(Hex)_1 (HexNAc)_2 (Fuc)_1 + (Man)_3(GlcNAc)_2$	1.7
2070.0395	$(Hex)_2 (HexNAc)_2 + (Man)_3(GlcNAc)_2$	1.3
2081.0537	$(HexNAc)_3 (Fuc)_1 + (Man)_3(GlcNAc)_2$	8.9
2173.0784	$(Hex)_2 (HexNAc)_1 (Fuc)_2 + (Man)_3(GlcNAc)_2$	0.0
2186.0854	$(Hex)_2 (HexNAc)_1 (NeuAc)_1 + (Man)_3(GlcNAc)_2$	0.6
2192.0854	$(Hex)_5 + (Man)_3(GlcNAc)_2$	3.4
2244.1277	$(Hex)_2 (HexNAc)_2 (Fuc)_1 + (Man)_3(GlcNAc)_2$	3.8
2285.1539	$(Hex)_1 (HexNAc)_3 (Fuc)_1 + (Man)_3(GlcNAc)_2$	1.1
2360.1746	$(Hex)_2 (HexNAc)_1 (Fuc)_1 (NeuAc)_1 + (Man)_3(GlcNAc)_2$	1.9
2390.1850	$(Hex)_3 (HexNAc)_1 (NeuAc)_1 + (Man)_3(GlcNAc)_2$	2.8
2396.1844	$(Hex)_6 + (Man)_3(GlcNAc)_2$	5.1
2418.2135	$(Hex)_2 (HexNAc)_2 (Fuc)_2 + (Man)_3(GlcNAc)_2$	1.2
2459.2430	$(Hex)_1 (HexNAc)_3 (Fuc)_2 + (Man)_3(GlcNAc)_2$	6.8
2489.2510	$(Hex)_2 (HexNAc)_3 (Fuc)_1 + (Man)_3(GlcNAc)_2$	0.6
2530.2780	$(Hex)_1 (HexNAc)_4 (Fuc)_1 + (Man)_3(GlcNAc)_2$	0.2
2564.2746	$(Hex)_3 (HexNAc)_1 (Fuc)_1 (NeuAc)_1 + (Man)_3(GlcNAc)_2$	2.4
2600.2838	$(Hex)_7 + (Man)_3(GlcNAc)_2$	0.2
2605.3014	$(Hex)_2 (HexNAc)_2 (Fuc)_1 (NeuAc)_1 + (Man)_3(GlcNAc)_2$	0.7
2622.3093	$(Hex)_3 (HexNAc)_2 (Fuc)_2 + (Man)_3(GlcNAc)_2$	0.3
2646.3256	$(Hex)_1 (HexNAc)_3 (Fuc)_1 (NeuAc)_1 + (Man)_3(GlcNAc)_2$	1.7
2704.3674	$(Hex)_1 (HexNAc)_4 (Fuc)_2 + (Man)_3(GlcNAc)_2$	0.5
2751.3607	$(Hex)_3 (HexNAc)_1 (NeuAc)_2 + (Man)_3(GlcNAc)_2$	0.1
2809.4016	$(Hex)_3 (HexNAc)_2 (Fuc)_1 (NeuAc)_1 + (Man)_3(GlcNAc)_2$	1.0
2837.4297	$(Hex)_2 (HexNAc)_3 (Fuc)_3 + (Man)_3(GlcNAc)_2$	1.4
2850.4258	$(Hex)_2 (HexNAc)_3 (Fuc)_1 (NeuAc)_1 + (Man)_3(GlcNAc)_2$	0.6
2966.4758	$(Hex)_2 (HexNAc)_2 (Fuc)_1 (NeuAc)_2 + (Man)_3(GlcNAc)_2$	5.3
2983.4875	$(Hex)_3 (HexNAc)_2 (Fuc)_2 (NeuAc)_1 + (Man)_3(GlcNAc)_2$	0.4
2996.4874	$(Hex)_3 (HexNAc)_2 (NeuAc)_2 + (Man)_3(GlcNAc)_2$	0.6
3024.5083	$(Hex)_2 (HexNAc)_3 (Fuc)_2 (NeuAc)_1 + (Man)_3(GlcNAc)_2$	0.9
3054.5327	$(Hex)_3 (HexNAc)_3 (Fuc)_1 (NeuAc)_1 + (Man)_3(GlcNAc)_2$	0.4
3082.5575	$(Hex)_2 (HexNAc)_4 (Fuc)_3 + (Man)_3(GlcNAc)_2$	1.2
3095.5615	$(Hex)_2 (HexNAc)_4 (Fuc)_1 (NeuAc)_1 + (Man)_3(GlcNAc)_2$	0.1
3170.5761	$(Hex)_3 (HexNAc)_2 (Fuc)_1 (NeuAc)_2 + (Man)_3(GlcNAc)_2$	8.1
3183.5907	$(Hex)_3 (HexNAc)_5 (Fuc)_1 + (Man)_3(GlcNAc)_2$	0.2
3200.5907	$(Hex)_4 (HexNAc)_2 (NeuAc)_2 + (Man)_3(GlcNAc)_2$	0.8
3269.6416	$(Hex)_2 (HexNAc)_4 (Fuc)_2 (NeuAc)_1 + (Man)_3(GlcNAc)_2$	0.4
3344.6614	$(Hex)_3 (HexNAc)_2 (Fuc)_2 (NeuAc)_2 + (Man)_3(GlcNAc)_2$	0.8
3374.6762	$(Hex)_4 (HexNAc)_2 (Fuc)_1 (NeuAc)_2 + (Man)_3(GlcNAc)_2$	3.9
3385.6846	$(Hex)_2 (HexNAc)_3 (Fuc)_2 (NeuAc)_2 + (Man)_3(GlcNAc)_2$	0.1
3387.6935	$(Hex)_4 (HexNAc)_5 (Fuc)_1 + (Man)_3(GlcNAc)_2$	0.5
3456.7318	$(Hex)_2 (HexNAc)_4 (Fuc)_1 (NeuAc)_2 + (Man)_3(GlcNAc)_2$	0.2
3514.7686	$(Hex)_2 (HexNAc)_5 (Fuc)_2 (NeuAc)_1 + (Man)_3(GlcNAc)_2$	0.1
3533.7472	$(Hex)_5 (HexNAc)_4 (NeuAc)_1 + (Man)_3(GlcNAc)_2$	1.1
3548.7659	$(Hex)_4 (HexNAc)_2 (Fuc)_2 (NeuAc)_2 + (Man)_3(GlcNAc)_2$	2.0
3589.7845	$(Hex)_3 (HexNAc)_3 (Fuc)_2 (NeuAc)_2 + (Man)_3(GlcNAc)_2$	0.2
3619.8082	$(Hex)_4 (HexNAc)_3 (Fuc)_1 (NeuAc)_2 + (Man)_3(GlcNAc)_2$	0.2
3776.8773	$(Hex)_3 (HexNAc)_3 (Fuc)_1 (NeuAc)_3 + (Man)_3(GlcNAc)_2$	0.6
4185.0789	$(Hex)_5 (HexNAc)_3 (Fuc)_1 (NeuAc)_3 + (Man)_3(GlcNAc)_2$	0.5
4400.1875	$(Hex)_4 (HexNAc)_4 (Fuc)_2 (NeuAc)_3 + (Man)_3(GlcNAc)_2$	0.4
4587.2797	$(Hex)_4 (HexNAc)_4 (Fuc)_1 (NeuAc)_4 + (Man)_3(GlcNAc)_2$	0.5
4948.4513	$(Hex)_4 (HexNAc)_4 (Fuc)_1 (NeuAc)_5 + (Man)_3(GlcNAc)_2$	0.4

Table S3. Relative abundancies of each of the N-glycans from the Mgat1b$^{-/-}$ strain.

Observed m/z (Na^+)	Composition	Relative %
1579.7839	$(Hex)_2 + (Man)_3(GlcNAc)_2$	30.1
1783.8842	$(Hex)_3 + (Man)_3(GlcNAc)_2$	7.3
1835.9242	$(HexNAc)_2 (Fuc)_1 + (Man)_3(GlcNAc)_2$	0.6
1987.9845	$(Hex)_4 + (Man)_3(GlcNAc)_2$	3.8
2040.0259	$(Hex)_1 (HexNAc)_2 (Fuc)_1 + (Man)_3(GlcNAc)_2$	1.4
2081.0530	$(HexNAc)_3 (Fuc)_1 + (Man)_3(GlcNAc)_2$	8.4
2186.0844	$(Hex)_2 (HexNAc)_1 (NeuAc)_1 + (Man)_3(GlcNAc)_2$	0.4
2192.0840	$(Hex)_5 + (Man)_3(GlcNAc)_2$	3.1
2244.1257	$(Hex)_2 (HexNAc)_2 (Fuc)_1 + (Man)_3(GlcNAc)_2$	2.6
2285.1524	$(Hex)_1 (HexNAc)_3 (Fuc)_1 + (Man)_3(GlcNAc)_2$	0.6
2360.1725	$(Hex)_2 (HexNAc)_1 (Fuc)_1 (NeuAc)_1 + (Man)_3(GlcNAc)_2$	0.9
2396.1837	$(Hex)_6 + (Man)_3(GlcNAc)_2$	3.6
2418.2080	$(Hex)_2 (HexNAc)_2 (Fuc)_2 + (Man)_3(GlcNAc)_2$	0.8
2459.2427	$(Hex)_1 (HexNAc)_3 (Fuc)_2 + (Man)_3(GlcNAc)_2$	5.3
2489.2513	$(Hex)_2 (HexNAc)_3 (Fuc)_1 + (Man)_3(GlcNAc)_2$	0.4
2564.2743	$(Hex)_3 (HexNAc)_1 (Fuc)_1 (NeuAc)_1 + (Man)_3(GlcNAc)_2$	1.8
2605.2994	$(Hex)_2 (HexNAc)_2 (Fuc)_1 (NeuAc)_1 + (Man)_3(GlcNAc)_2$	1.1
2646.3221	$(Hex)_1 (HexNAc)_3 (Fuc)_1 (NeuAc)_1 + (Man)_3(GlcNAc)_2$	1.0
2809.3995	$(Hex)_3 (HexNAc)_2 (Fuc)_1 (NeuAc)_1 + (Man)_3(GlcNAc)_2$	1.0
2837.4269	$(Hex)_2 (HexNAc)_3 (Fuc)_3 + (Man)_3(GlcNAc)_2$	0.9
2850.4221	$(Hex)_2 (HexNAc)_3 (Fuc)_1 (NeuAc)_1 + (Man)_3(GlcNAc)_2$	0.4
2966.4748	$(Hex)_2 (HexNAc)_2 (Fuc)_1 (NeuAc)_2 + (Man)_3(GlcNAc)_2$	4.2
2996.4853	$(Hex)_3 (HexNAc)_2 (NeuAc)_2 + (Man)_3(GlcNAc)_2$	0.8
3024.5031	$(Hex)_2 (HexNAc)_3 (Fuc)_2 (NeuAc)_1 + (Man)_3(GlcNAc)_2$	0.6
3054.5302	$(Hex)_3 (HexNAc)_3 (Fuc)_1 (NeuAc)_1 + (Man)_3(GlcNAc)_2$	0.4
3082.5574	$(Hex)_2 (HexNAc)_4 (Fuc)_3 + (Man)_3(GlcNAc)_2$	0.4
3095.5583	$(Hex)_2 (HexNAc)_4 (Fuc)_1 (NeuAc)_1 + (Man)_3(GlcNAc)_2$	0.1
3140.5490	$(Hex)_2 (HexNAc)_2 (Fuc)_2 (NeuAc)_2 + (Man)_3(GlcNAc)_2$	0.6
3170.5752	$(Hex)_3 (HexNAc)_2 (Fuc)_1 (NeuAc)_2 + (Man)_3(GlcNAc)_2$	6.9
3200.5856	$(Hex)_4 (HexNAc)_2 (NeuAc)_2 + (Man)_3(GlcNAc)_2$	1.1
3211.5953	$(Hex)_2 (HexNAc)_3 (Fuc)_1 (NeuAc)_2 + (Man)_3(GlcNAc)_2$	0.6
3344.6561	$(Hex)_3 (HexNAc)_2 (Fuc)_2 (NeuAc)_2 + (Man)_3(GlcNAc)_2$	0.8
3374.6761	$(Hex)_4 (HexNAc)_2 (Fuc)_1 (NeuAc)_2 + (Man)_3(GlcNAc)_2$	2.8
3387.6880	$(Hex)_4 (HexNAc)_5 (Fuc)_1 + (Man)_3(GlcNAc)_2$	0.2
3415.6970	$(Hex)_3 (HexNAc)_3 (Fuc)_1 (NeuAc)_2 + (Man)_3(GlcNAc)_2$	0.6
3533.7391	$(Hex)_5 (HexNAc)_4 (NeuAc)_1 + (Man)_3(GlcNAc)_2$	1.7
3548.7633	$(Hex)_4 (HexNAc)_2 (Fuc)_2 (NeuAc)_2 + (Man)_3(GlcNAc)_2$	2.4
3776.8770	$(Hex)_3 (HexNAc)_3 (Fuc)_1 (NeuAc)_3 + (Man)_3(GlcNAc)_2$	0.4
4587.2754	$(Hex)_4 (HexNAc)_4 (Fuc)_1 (NeuAc)_4 + (Man)_3(GlcNAc)_2$	0.2
4948.4490	$(Hex)_4 (HexNAc)_4 (Fuc)_1 (NeuAc)_5 + (Man)_3(GlcNAc)_2$	0.2

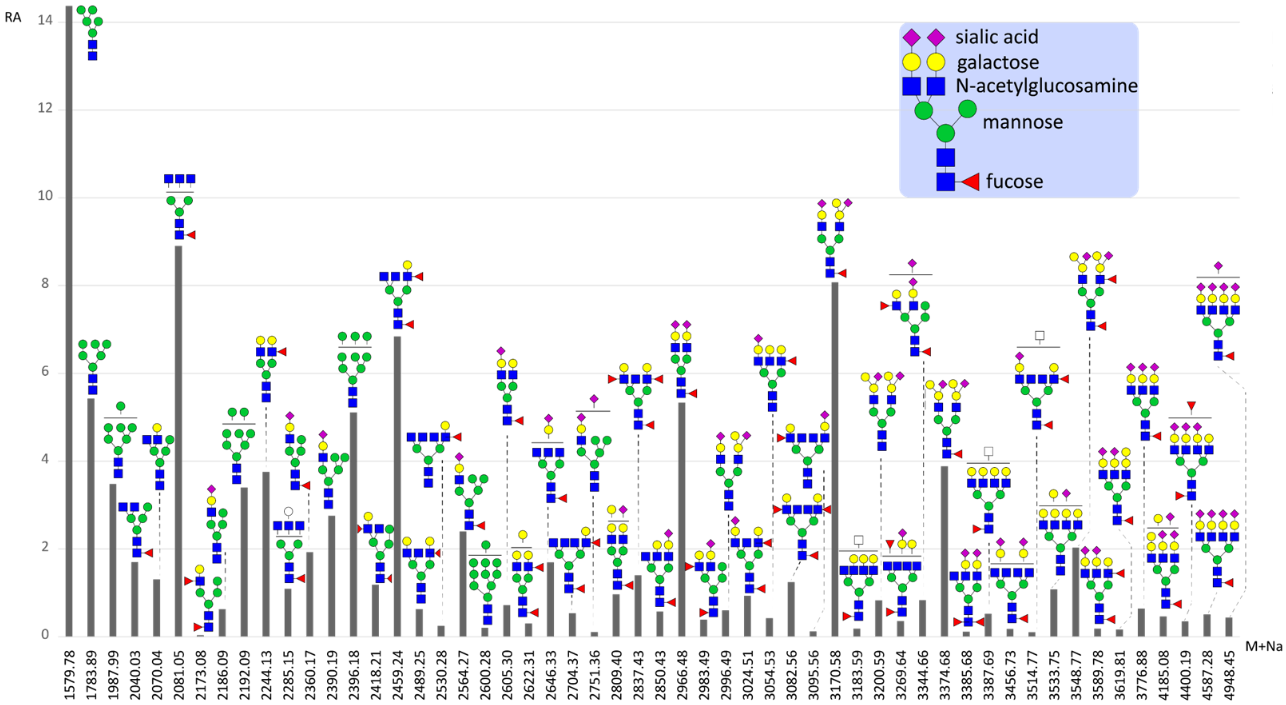

Figure S1. LC-ESI-MS intensities of the permethylated N-glycans derived from the Wt AB zebrafish strain.

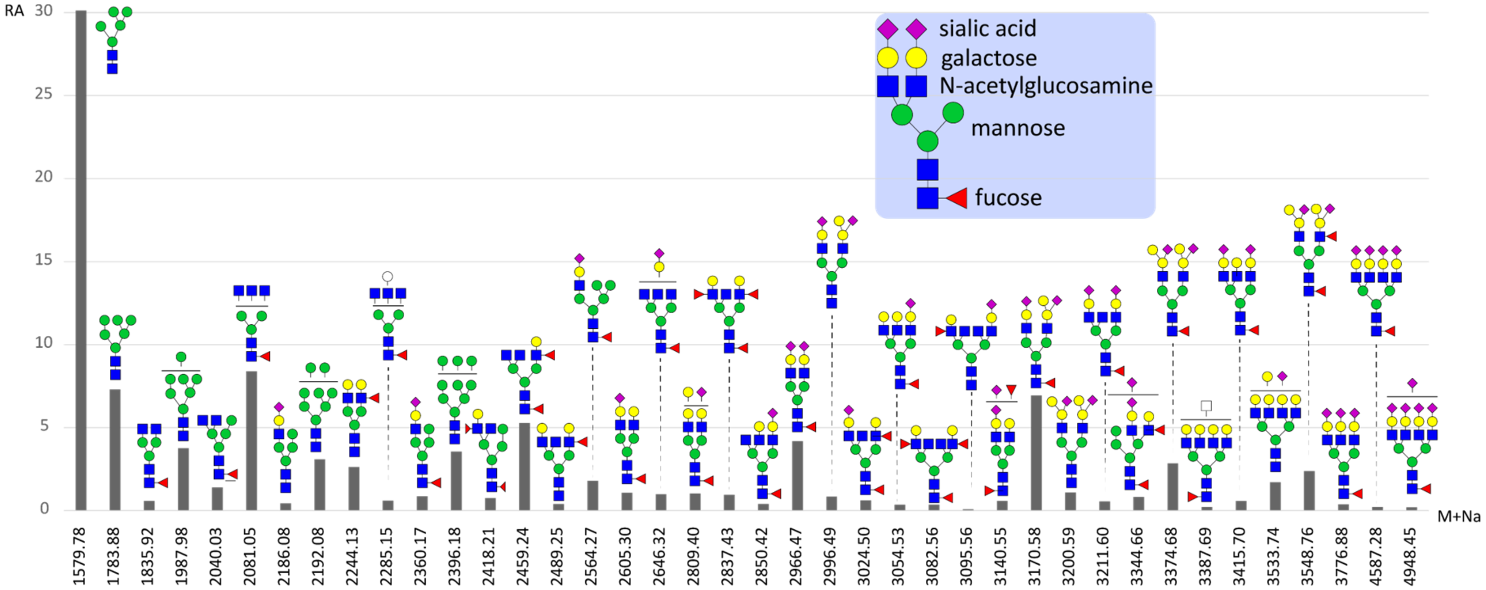

Figure S2. LC-ESI-MS intensities of the permethylated N-glycans derived from the Mgat1b^{-}/$^{-}$zebrafish strain.

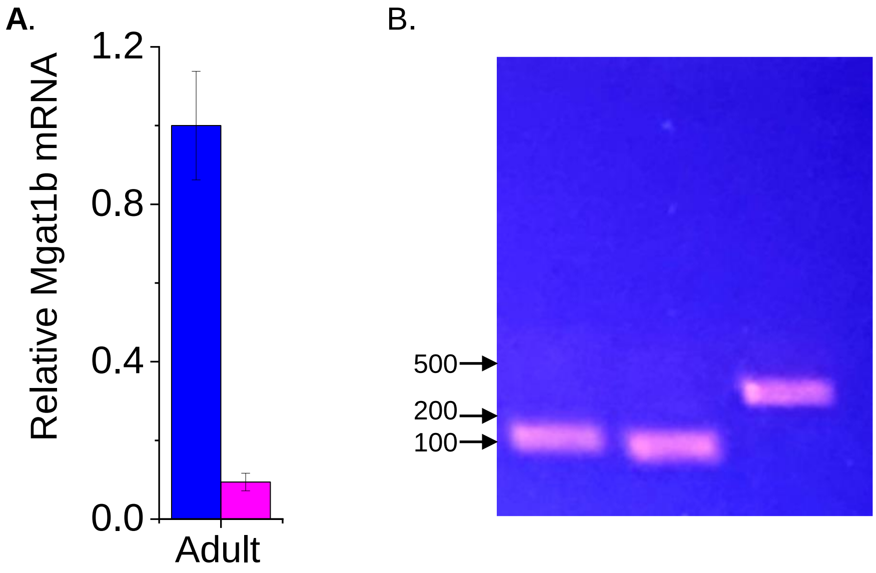

Figure S3. Comparing relative levels of Mgat1b transcripts from adult Wt AB and Mgat1b$^{-/-}$ zebrafish strains. Transcript levels were normalized to elfa (A). RT-PCR products run on agarose gel (B). The sizes of the fragments were as follows: Mgat1b, 129 bp; β-actin, 95 bp; elfa, 358 bp.

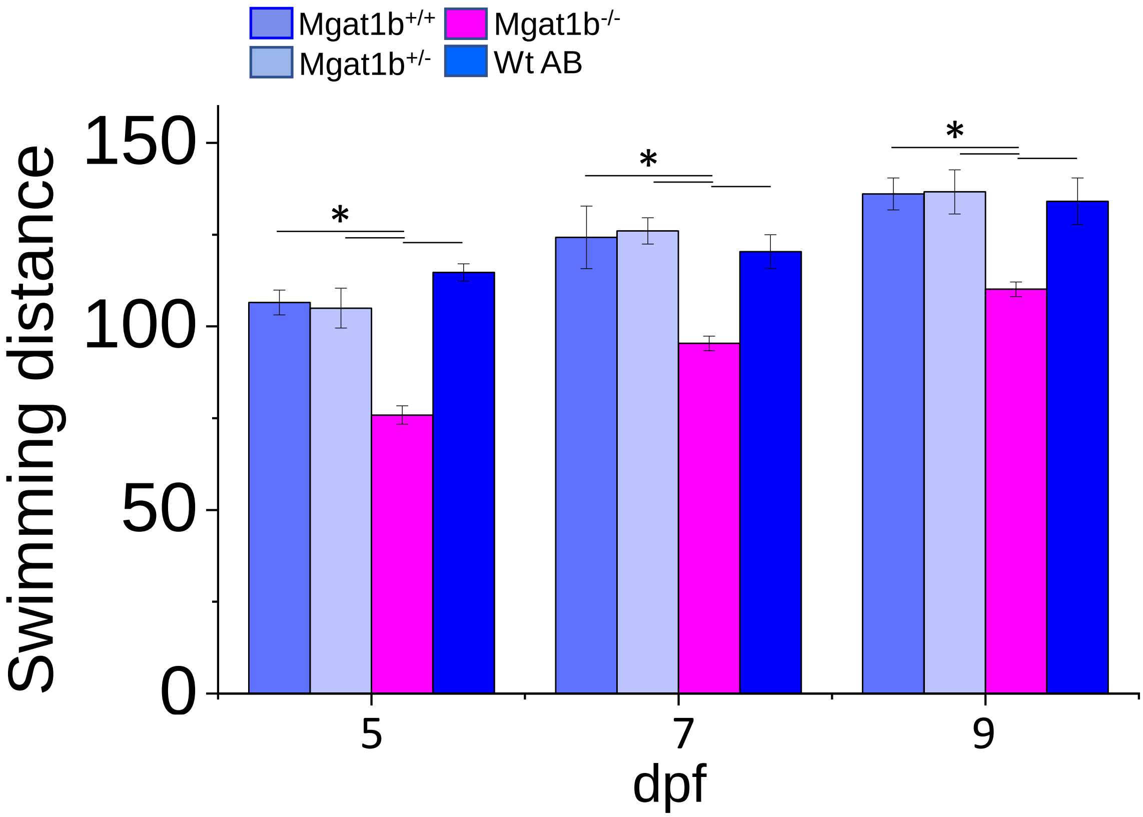

Figure S4. Swimming locomotor activity of Wt AB and the various Mgat1b zebrafish strains. Data are presented as mean ± SEM,n≥21andwerecomparedbyone-way ANOVA with Holmes-Bonferroni adjustments, $^*p < 0.05$).
